# Success of Strabismus Surgery in Intermittent Exotropia

**DOI:** 10.3390/jpm15080333

**Published:** 2025-07-30

**Authors:** Pedro Lino, Pedro Vargues de Aguiar, João Paulo Cunha

**Affiliations:** 1 NOVA National School of Public Health, Public Health Research Centre, Comprehensive Health Research Center, CHRC, LA-REAL, CCAL, NOVA University Lisbon, 1649-004 Lisbon, Portugal; pedroaguiar@ensp.unl.pt; 2 Santa Maria Local Health Unit, 1649-028 Lisbon, Portugal; 3 CUF Cascais Hospital, 2750-663 Cascais, Portugal; 4 CUF Tejo Hospital, 1350-352 Lisbon, Portugal; 5 Higher School of Technology and Health of Lisbon, Polytechnic Institute of Lisbon, 1990-096 Lisbon, Portugal; 6 Ophthalmology Unit, CUF Cascais Hospital, 2750-663 Cascais, Portugal; joao.cunha@estesl.ipl.pt; 7 Department of Morpho-Functional Sciences, Higher School of Technology and Health of Lisbon, Polytechnic Institute of Lisbon, 1990-096 Lisbon, Portugal

**Keywords:** intermittent exotropia, surgery, surgical success, recurrence

## Abstract

**Introduction**: Intermittent exotropia (IXT) is the most common form of childhood divergent strabismus. Surgery remains the primary approach to control ocular deviation and preserve binocular function. Although previous studies report a success rate of approximately 75%, factors influencing surgical outcomes remain insufficiently explored. This study evaluates the effectiveness of strabismus surgery in children with IXT and identifies predictors of postoperative alignment stability. **Methods**: This retrospective study included 258 children with basic-type IXT or divergence excess who underwent bilateral lateral rectus recession. Clinical records and surgical data were analyzed to determine the overall success rate and identify associated predictive factors. **Results**: The sample included 166 females (64.3%) and 92 males (35.7%), with a mean age of 11.19 ± 3.73 years. Surgical success was achieved in 238 patients (92.2%). Success rates were similar across sexes (92.8% in females vs. 91.3% in males). No significant associations were found between surgical success and sex, age, preoperative occlusion therapy, binocular function, or IXT subtype. However, patients with moderate preoperative deviations had higher success rates. A statistically significant difference was observed in the preoperative deviation angle between successful (31 ± 7.08∆) and unsuccessful (42 ± 7.27∆) cases. A lower AC/A ratio was also associated with better outcomes, although it was not the main predictor. **Discussion**: The high success rate (92.2%) suggests a limited impact of demographic or preoperative variables. The preoperative deviation angle emerged as the strongest predictor of success, with smaller angles correlating with more favorable surgical outcomes. These findings underscore the importance of accurate preoperative assessment in surgical planning for IXT.

## 1. Introduction

Intermittent exotropia (IXT) is the most common type of divergent strabismus in children. It is defined as a deviation that is present intermittently and alternates with periods of orthotropia [1,2,3]. The deviation usually appears during early childhood (between 1 and 5 years of age) and typically becomes more frequent over time. It often manifests during distance fixation or in situations of fatigue, distraction, or illness. As the child grows, the deviation may also appear at near fixation and can eventually progress to constant exotropia (XT) [1]. The development of amblyopia is rare and usually occurs only when the condition becomes constant at an early age or when it coexists with other amblyogenic factors, such as uncorrected refractive errors [1].

Typically, during periods of ocular alignment, individuals exhibit binocular vision and stereopsis. When the deviation is present, anomalous retinal correspondence (ARC) may occur. Depending on the patient’s age and the maturity of the visual system, suppression or diplopia may develop [1,2,3,4,5].

According to Duane’s classification, IXT can be divided into four types: divergence excess, basic type, convergence insufficiency, and pseudo-divergence excess [1,5].

There is currently no universally accepted first-line treatment for IXT [1]. Treatment options include medical approaches, such as occlusion therapy and the use of minus lenses to stimulate convergence, and orthoptic therapy, consisting of fusion exercises, which remain controversial due to ongoing debate regarding their effectiveness and their potential negative impact on surgical outcomes. Orthoptic therapy may be useful only in cases of convergence insufficiency, as it can promote fixation and improve fusion. Therefore, surgical intervention is the mainstay of treatment for most IXT cases. Surgery typically yields satisfactory results, although undercorrection or overcorrection may occur in the immediate postoperative period. Over time, XT may recur, and additional surgery may be required [1,4,5,6,7]. Surgery for IXT has reported success rates of 70–80%, but recurrence can occur in up to 50% of cases within three years. Several prognostic factors strongly influence outcomes [8,9,10,11,12,13,14]. A smaller preoperative deviation angle is associated with better surgical success and a reduced need for reoperation [5,8,10,15]. Younger age at the time of surgery, particularly between 3 and <5 years, is also linked to higher success rates [8,14]. The type of exotropia is another important factor: the pseudo-divergence excess subtype is associated with better alignment and lower recurrence [14,15,16]. Postoperative alignment during the first week is a strong predictor of long-term success, especially in adults. In cases with large deviation angles (>60 PD), the likelihood of achieving orthophoria is high, although binocular success may be limited [5,9]. A family history of strabismus surgery does not appear to significantly influence surgical outcomes [4].

This study aims to evaluate the effectiveness of bilateral lateral rectus recession surgery in children with intermittent exotropia (IXT), with a particular focus on identifying predictive factors for long-term alignment stability. In contrast to previous studies, this research provides a comprehensive analysis of a large pediatric cohort in Portugal, applying a personalized medicine approach to stratify surgical outcomes based on clinical profiles such as preoperative deviation angle and AC/A ratio.

## 2. Methodology

This was a retrospective study [17].of patients treated at CUF Cascais Hospital, involving a cohort of children who underwent strabismus surgery for intermittent exotropia. The procedure performed was classic bilateral lateral rectus recession under general anesthesia. All surgeries were conducted by the same surgeon, and the surgical dose was determined based on the distance deviation angle using Park’s formula (Table 1).

Inclusion criteria: Children aged 4 to 18 years with a confirmed diagnosis of intermittent exotropia who underwent surgical correction. Only patients with complete medical records—including preoperative data, surgical reports, and postoperative follow-up—were included. All participants had normal visual acuity (i.e., no amblyopia) and no associated ocular or systemic conditions that could influence the evaluation of surgical outcomes.

Exclusion criteria: Patients were excluded if they presented other ocular or systemic pathologies, unilateral or bilateral amblyopia, chromosomal abnormalities or systemic diseases (e.g., congenital anomalies or neurological disorders), paralytic or restrictive exotropia, dissociated vertical deviation or alphabet patterns, constant exotropia, or other types of divergent strabismus such as consecutive or sensory exotropia.

The following parameters were documented and analyzed: sex, age, age at surgery, refractive error (spherical equivalent), distance deviation in prism diopters (PD) before surgery, preoperative sensory status, type of surgical procedure performed, postoperative deviation in PD at six months, and sensory function at six months. A six-month follow-up was chosen because ocular alignment is generally more stable after this period, allowing for a more accurate assessment of residual deviation. This timeframe also permits evaluation of sensory adaptation (e.g., binocular fusion), identification of relapses or overcorrections, and assessment of symptoms and quality of life. Moreover, it is a commonly used interval in scientific literature, facilitating comparison with other studies.

In all patients, deviations were measured using the alternate cover test with prisms for both near (33 cm) and distance (6 m) fixation. To differentiate between true divergence excess and pseudo-divergence excess, measurements were repeated after occlusion (1 h) of the non-dominant eye. The AC/A ratio was calculated using the gradient method, with the addition of −2.00 D lenses.

Sensory status was evaluated using fusion (Worth 4-dot test) and stereopsis (Wirt test for near, Vectograph for distance).

Definition of surgical success: Residual ocular deviation ≤10 PD at distance fixation six months after surgery (whether eso- or exotropic), with the presence of sensory fusion and stereopsis of 80 arcseconds or better, as assessed by either near (Wirt) or distance (Vectograph) stereotests. This combined motor and sensory criterion aligns with prior literature and enables a comprehensive evaluation of functional surgical outcomes.

Statistical analysis [18,19]: Data were entered into a custom SPSS^®^ version 29 database and analyzed using univariate, bivariate, and multivariate statistical methods. Variables were classified according to their characteristics and categorized accordingly.

Qualitative sociodemographic and clinical variables were analyzed using descriptive statistics, and quantitative variables were summarized using measures of central tendency and dispersion. Logistic regression was used to identify factors associated with stable postoperative alignment, including both crude and adjusted models. Variables included in the model were selected based on clinical relevance and statistical significance in bivariate analysis, and multicollinearity was assessed to ensure model robustness. A significance level of 5% was adopted, and *p*-values below this threshold were considered statistically significant.

All stages of the research complied with ethical and regulatory standards for Good Practice in Health Research and were approved by the Ethics Committee of the CUF Group—Health Services (approval number: 2024/488).

## 3. Results

The sample included 258 participants: 166 females (64.3%) and 92 males (35.7%). The participants’ ages ranged from 5 to 18 years, with a mean age of 11.19 ± 3.73 years.

The mean age at the time of surgery was 8.75 ± 12.19 years, with a minimum of 4 years and a maximum of 17 years.

Out of the total sample, 238 patients (92.2%) achieved surgical success, defined as a residual eso- or exodeviation ≤10 PD and stereopsis better than 80 arcseconds. The remaining 20 patients (7.8%) did not meet the criteria for success. In the success group, the mean refractive error (spherical equivalent) was −0.63 ± 1.424 D, while in the no-success group, it was −0.63 ± 1.134 D.

Ophthalmologic characteristics: Table 2 shows the associations between each of the characteristics studied in each of the groups (success vs. non-success).

- Preoperative occlusive treatment: 196 patients (91.2%) underwent occlusion therapy as an anti-suppression exercise.
- Binocular function before surgery: 233 patients (92.5%) had binocular function prior to surgery. The odds ratio (OR) for surgical success in patients with binocular vision was 2.453 (*p* = 0.424).
- IXT type: 187 patients (93%) had basic-type intermittent exotropia, and 51 patients (89.5%) had divergence excess type. The OR for surgical success in patients with basic-type IXT was 0.636 (*p* = 0.378).
- Preoperative deviation angle: The mean deviation at distance was 31 ± 7.08∆ in the success group and 42 ± 7.27∆ in the no-success group. Patients with moderate deviation had a significantly higher chance of surgical success.
- AC/A ratio: The mean AC/A ratio was 7.09 ± 3.735 in the success group and 11.27 ± 9.035 in the no-success group. A statistically significant difference was found (OR = 0.920; *p* = 0.013), suggesting that a higher AC/A ratio is associated with lower surgical success.
- Total surgical dose: The mean total amount of recession (bilateral) was 13.65 ± 2.5 mm in the success group and 15.33 ± 3.395 mm in the no-success group. Although there was no statistically significant difference between larger setbacks (t = 1.891; *p* = 0.078), logistic regression showed a significant association (*p* = 0.025).
- Sex: Surgical success was achieved in 154 females (92.8%) and 84 males (91.3%). Although females tended to have a slightly higher success rate, the difference was not statistically significant (OR = 0.818; *p* = 0.673).
- Age and age at surgery: The mean age in the success group was 11.22 ± 3.589 years, compared to 12.20 ± 5.414 years in the no-success group. The mean age at surgery was 8.76 ± 12.617 years in the success group and 9.80 ± 5.519 years in the no-success group. There was no significant association between these variables and surgical success, although younger patients tended to have better outcomes (OR = 1.031; *p* = 0.627 for age; OR = 1.001; *p* = 0.954 for surgical age).

By further analyzing associations and interactions between variables using binary logistic regression, it was found that the preoperative deviation angle was the only exposure significantly influencing surgical outcome.

In the optimized logistic regression model (which included variables with *p* < 0.2 and excluded those with adjusted *p* > 0.05 after testing for collinearity), the only statistically significant predictor of surgical success was the preoperative deviation angle: patients with smaller deviation angles had a significantly higher probability of successful outcomes (OR = 0.837; *p* < 0.001).

## 4. Discussion

The primary objective of surgical intervention in intermittent exotropia (IXT) is to improve ocular alignment while preserving or enhancing binocular function. Therefore, surgical decisions are generally based on the clinical characteristics and functional impact of the strabismus. Large-angle exodeviations associated with reduced fusional amplitudes and a tendency toward loss of binocularity are typical indications for surgery.

### 4.1. Surgical Outcomes and Success Rates:

This study included 258 patients—166 females (64.3%) and 92 males (35.7%)—with ages ranging from 5 to 18 years (mean: 11.19 ± 3.73 years). The mean age at the time of surgery was 8.75 ± 12.19 years, with a minimum of 4 years and a maximum of 17 years. Surgical success was achieved in 238 patients (92.2%), while 20 patients (7.8%) did not achieve the defined criteria for success.

The overall surgical success rate in this study—exceeding 90%—is higher than what has been reported in several previous studies. For example, Donahue et al. reported a success rate of approximately 70% in a cohort of 197 patients in Florida [20]. This study represents the first publication from Portugal addressing bilateral surgery for IXT. Although a previous study with a small sample size reported a 100% success rate [4], our findings are based on a larger and more representative cohort. Reported success rates in the literature vary depending on the follow-up period. Kopmann et al. reported a success rate of 83.8% on the first postoperative day [5], while Thorisdottir et al. reported an 80% success rate at two months postoperatively [21]. In this context, our success rate at six months supports the high efficacy of bilateral lateral rectus muscle recession for IXT.

### 4.2. Factors Influencing Surgical Outcomes:

- Preoperative deviation angle: Statistical analysis showed that a smaller preoperative deviation angle was associated with higher surgical success. The mean angle in the success group was 31 ± 7.08∆, compared to 42 ± 7.27∆ in the no-success group. Patients with moderate deviations were significantly more likely to achieve success (OR = 9.259; *p* = 0.01). Quantitative analysis further confirmed the protective role of a smaller angle (OR = 0.837; *p* < 0.001), indicating that each additional prism diopter increased the risk of failure by approximately 16%. These results are consistent with previous findings, including those of Kopmann et al. (2024) [5], Tibrewal (2017) [22], and Thorisdottir (2022) [21], all of which suggest that patients with smaller preoperative angles have better outcomes.
- Age at surgery: The mean age in the success group was 11.22 ± 3.59 years, compared to 12.20 ± 5.41 years in the no-success group. The mean surgical age was 8.76 ± 12.62 years in the success group and 9.80 ± 5.52 years in the no-success group. Although no statistically significant association was found (*p* = 0.627 for age; *p* = 0.954 for surgical age), there was a tendency toward better results in younger patients. This is in agreement with Issaho et al. (2017) [11], although some authors, such as Jiménez-Romo (2023), argue that age is not a significant predictor of surgical success [23].
- Binocular function: Preoperative binocular function was present in 233 patients (92.5%). Although a tendency toward better outcomes in these patients was observed (OR = 2.453; *p* = 0.424), the association was not statistically significant. This suggests that surgery may either preserve or restore binocular function, depending on the preoperative status.
- AC/A ratio: The mean AC/A ratio was 7.09 ± 3.73 in the success group and 11.27 ± 9.04 in the no-success group. A higher AC/A ratio was significantly associated with lower surgical success (OR = 0.920; *p* = 0.013), indicating that patients with greater accommodative convergence relative to accommodation are less likely to benefit from surgery [24].
- Gender: Surgical success was achieved in 154 female patients (92.8%) and 84 male patients (91.3%). Although there was a slight trend toward higher success in females (OR = 0.818; *p* = 0.673), the difference was not statistically significant and may be due to chance.
- Preoperative occlusion therapy: Occlusion therapy was performed in 196 patients (91.2%). While it appeared to be a potential protective factor (OR = 0.246; *p* = 0.177), the association was not statistically significant. This finding is in line with the anterior works [14], who suggested that children undergoing occlusion therapy before surgery may have better outcomes.
- Surgical dose: The mean bilateral recession was 13.65 ± 2.5 mm in the success group and 15.33 ± 3.39 mm in the no-success group. Although the direct comparison did not reach statistical significance (*p* = 0.078), logistic regression revealed a significant negative association between larger recession amounts and surgical success (OR = 0.817; *p* = 0.025), indicating that higher surgical doses may be associated with a lower likelihood of favorable outcomes.

### 4.3. Study Limitations:

This study has several limitations, primarily related to its retrospective design. Patients were not randomly assigned to treatment protocols; instead, the surgical approach was based on the clinical judgment and experience of the operating surgeon. This may introduce selection bias and limit the generalizability of our findings. Nevertheless, the large sample size and comprehensive analysis strengthen the conclusions and provide meaningful insights into the factors influencing surgical success in IXT.

## 5. Conclusions

Children diagnosed with the basic type of intermittent exotropia tended to achieve more favorable surgical outcomes. After adjusting for potential confounding variables, a statistically significant association was found between smaller preoperative deviation angles and a greater likelihood of surgical success.

Although younger age at the time of surgery and the presence of preoperative binocular function did not reach statistical significance, these factors may still hold clinical relevance and should be considered during surgical planning and decision-making.

## Figures and Tables

**Table 1:** Parks’ formula for exotropia surgery.

Exotropia Angle in PD	Amount of Recession/Resection in Mm	
Two muscles	BLRc	LRc + MRs (R&R)
15	4.0	4.0 + 3.0
20	5.0	5.0 + 4.0
25	6.0	6.0 + 4.0
30	7.0	7.0 + 5.5
35	7.5	7.5 + 5.5
40	8.0	8.0 + 5.5
45	8.5	8.5 + 6.0
50	9.0	9.0 + 6.5
60	10	9.0 + 7.0

Legend: PD, prismatic diopters. BLRc, bilateral lateral rectus recession. LRc, lateral rectus recession. MRs: medial rectus resection.

**Table 2:** Association between exposures and outcome.

Exposure	Group: Success	Group: No Success	OR	*p*-Value	95% Confidence Interval
Gender Female (reference) Male	154 (92.8%) 84 (91.3%)	12 (7.2%) 8 (8.7%)	0.818	0.673	0.322; 2.080
Age	11.22 ± 3.589	12.20 ± 5.414	1.031	0.627	0.911; 1.168
Surgical Age	8.76 ± 12.617	9.80 ± 5.519	1.001	0.954	0.962; 1.042
Prior occlusive treatment Yes No (reference)	196 (91.2%) 42 (97.7%)	19 (8.8%) 1 (2.3%)	0.246	0.177	0.032; 1.886
XTI type DET BT (reference)	51 (89.5%) 187 (93%)	6 (10.5%) 14(7%)	0.636	0.378	0.233; 1.739
Moderate preoperative deviation Yes No (reference)	76 (100%) 162 (89%)	0(0%) 20 (11%)	9.259 *	0.01	1.648; 196.3
Magnitude of preoperative deviation	31 ± 7.080	42 ± 7.270	0.837	<0.001	0.777; 0.901
AC/A ratio	7.09 ± 3.735	11.27 ± 9.035	0.920	0.013	0.861; 0.983
Binocular Preoperative Yes No (reference)	233 (92.5%) 5 (83.3%)	19 (7.5%) 1 (16.7%)	2.453	0.424	0.272; 22.077
Surgical Dose (mm)	13.65 ± 2.5	15.33 ± 3.395	0.817	0.025	0.685; 0.975

* For this calculation, the authors do a direct substitution of 0 by 1 OR (odds ratio).

## Data Availability

The raw data supporting the conclusions of this article will be made available by the authors on request.
